# Orthopedic referral rates following osteoporosis screening using dental panoramic radiography in female patients: A three-year prospective study

**DOI:** 10.1016/j.afos.2025.10.001

**Published:** 2025-10-06

**Authors:** Noriyuki Sugino, Hiroko Kuroiwa, Hizuru Osanai, Shinichiro Yamada, Kozue Mori, Hirokazu Kobayashi, Daisuke Higuchi, Nobuyuki Udagawa, Akira Taguchi

**Affiliations:** a Department of Oral and Maxillofacial Radiology, Matsumoto Dental University, Nagano, Japan; b Department of Pediatric Dentistry, School of Dentistry, Matsumoto Dental University, Nagano, Japan; c Department of Orthopedic Surgery, Matsumoto Dental University Hospital, Nagano, Japan; d Department of Prosthodontics, School of Dentistry, Matsumoto Dental University, Nagano, Japan; e Department of Biochemistry, School of Dentistry, Matsumoto Dental University, Nagano, Japan

**Keywords:** Education, Mass screening, Orthopedics, Osteoporosis, Radiography, Panoramic

## Abstract

**Objectives:**

Although osteoporosis affects approximately 15.9 million people in Japan, screening rates remain low. Dental panoramic radiographs (DPRs), routinely used in general dental practice, may facilitate opportunistic screening, yet referrals based on these images are not widely implemented. This study evaluated referral rates to orthopedic departments of patients with suspected osteoporosis identified using DPRs.

**Methods:**

Among 3237 female patients aged ≥ 50 who underwent DPRs between February 2022 and October 2024, 328 without a prior osteoporosis diagnosis were identified as suspected cases. Their primary dentists referred these patients to our hospital's orthopedic department. Additionally, trained oral radiologists optionally provided information on osteoporosis. We assessed (1) the overall referral rate, (2) differences in referral rates based on whether an explanation by oral radiologist was provided, (3) referral rates stratified by age group, and (4) the prevalence of osteoporosis, osteopenia, and asymptomatic vertebral fractures among referred patients.

**Results:**

Of the 328 patients, 53 (16.2%) visited the orthopedic department. Referral rates were significantly higher among patients who received explanations from trained oral radiologists (50.9%) than among patients who did not (9.1%). Referral rates peaked among patients in their 60s (23.7%). Among referred patients, 60.4% were diagnosed with osteoporosis and 37.7% with osteopenia; five patients had asymptomatic vertebral fractures.

**Conclusions:**

Despite easy access to orthopedic care, referral rates remained low, likely due to limited awareness. Explanations by trained oral radiologists significantly improved referral rates, highlighting the importance of dentist-led education and interdisciplinary collaboration in promoting osteoporosis screening in general dental practice.

## 1 Introduction

Fragility fractures associated with osteoporosis, particularly in postmenopausal women, impose substantial societal burdens, including increased mortality, reduced quality of life, and significant economic costs [1]. Patients with both hip and concomitant vertebral fractures have a significantly higher risk of mortality than those without such fractures [2]. Severe vertebral fractures, but not mild vertebral fractures, are independently associated with low back pain and impaired mobility [3]. Given that vertebral fractures are often asymptomatic, early identification of postmenopausal women at risk for osteoporosis is essential. In Denmark, a two-step population-based osteoporosis screening program utilizing the Fracture Risk Assessment Tool was shown to reduce the incidence of hip fractures, overall fractures, and mortality among older women [4]. At approximately 5.5%, the average rate of attendance for medical checkups in Japan remains considerably low [5], leaving a substantial proportion of postmenopausal women with undiagnosed osteoporosis and at risk of developing fragility fractures.

Dental panoramic radiographs (DPRs) are widely used to assess dental lesions such as dental caries, chronic periodontitis, and periapical diseases. Dentists also use DPRs to diagnose various oral and maxillofacial conditions, including osteomyelitis, benign and malignant tumors, and temporomandibular joint disorders. Annually, approximately 5 million DPRs are captured in individuals aged 65 years and older in Japan [6]. The utility of DPRs in identifying postmenopausal women at risk for osteoporosis has also been investigated. The morphology of the mandibular inferior cortex, as observed on DPRs, correlates with bone mineral density (BMD) of the lumbar spine and femoral neck [7,8], biochemical markers of bone turnover [9,10], and risk of fragility fractures [11,12]. A recent systematic review and meta-analysis concluded that the mandibular radiomorphometric indices observed on DPRs are useful as a screening tool for detecting osteoporosis in postmenopausal women [13].

In 2021, the Japanese Society for Oral and Maxillofacial Radiology (JSOMR) released clinical guidelines endorsing panoramic radiographic measurements in general dental practice to identify asymptomatic postmenopausal women at risk for osteoporosis [14]. Concurrently, JSOMR consulted the Japan Osteoporosis Society and the Japanese Orthopedic Association on referral pathways for suspected cases; both societies agreed to accept such referrals. Since then, some dentists in Japan have begun referring women with DPR-identified risk directly for medical evaluation [15].

To assess the feasibility of these referrals, we conducted a pilot study in 2007 [16]. In Hiroshima Prefecture, general dentists identified postmenopausal women at risk, but only a small proportion underwent BMD assessments at the university hospital. Of those who underwent dual-energy X-ray absorptiometry (DXA), most had low bone mass, and a substantial proportion had asymptomatic vertebral fractures. These results highlighted both the potential value of dental screening and the barriers to successful referral, leading us to design the present study. Whether these women subsequently sought osteoporosis treatment after BMD assessment remains unclear.

To date, little is known about referral rates to orthopedic care for patients identified as at risk of osteoporosis through dental panoramic radiography in general practice. Therefore, this prospective study aimed to investigate these referral rates at our dental university hospital and to evaluate whether providing patients with information about osteoporosis influenced referral.

## 2 Methods

### 2.1 Participants and imaging protocol

Female patients aged ≥ 50 who underwent DPRs for dental treatment at Matsumoto Dental University Hospital between February 2022 and October 2024 and who were suspected of having osteoporosis based on mandibular inferior cortex morphology on DRPs were eligible for this prospective study. Female patients with a history of orthopedic consultation for osteoporosis were excluded. This study focused exclusively on women, as evidence for panoramic radiographic screening in men remains limited [14]. Suspected cases were referred to our Department of Orthopedics for further evaluation, including lateral spine radiography, DXA, and biochemical markers of bone turnover.

DPRs were taken using a Hyper-X unit (Asahi Roentgen Ind. Co., Kyoto, Japan) under the following conditions: tube voltage, 64 kV; tube current, 8 mA; and exposure time, 12 s (180° rotation). The images were processed using a computed radiography system (Regius 170; Konica Minolta, Inc., Tokyo, Japan) and interpreted on a diagnostic monitor (PGL21; WIDE Corporation, Seoul, South Korea).

### 2.2 Classification of the mandibular inferior cortex

**Fig. 1:**
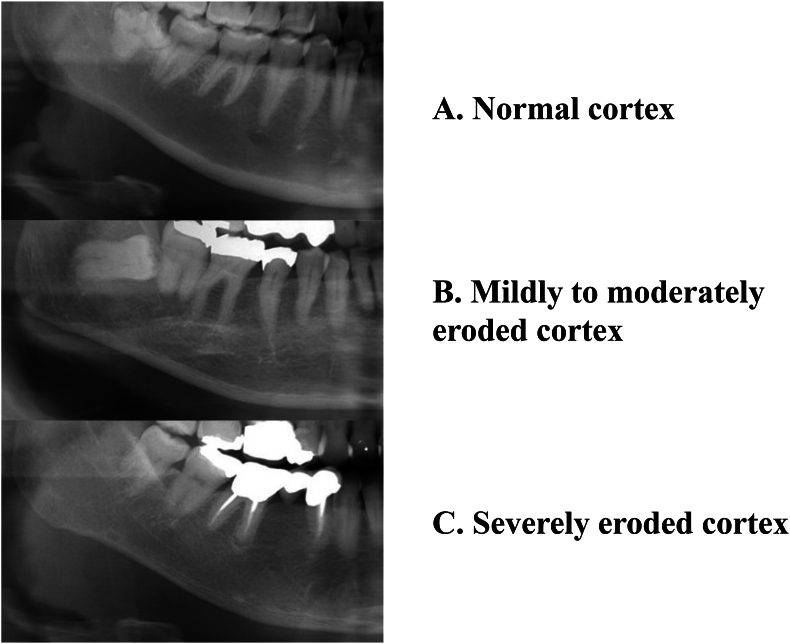
Classification of mandibular inferior cortical morphology on dental panoramic radiographs: **A.** Normal cortex with an even, sharp endosteal margin on both sides. **B.** Mildly to moderately eroded cortex with semilunar endosteal defects and residual cortical fragments. **C.** Severely eroded cortex with pronounced porosity and substantial endosteal cortical residues.

Using the DPRs, the mandibular inferior cortex was classified into three categories (Fig. 1) [11]: Normal cortex: the endosteal margin was even and sharp on both sides (Fig. 1A); Mildly to moderately eroded cortex: the endosteal margin showed semilunar defects (lacunar resorption) or endosteal cortical residues appeared to have formed (Fig. 1B); Severely eroded cortex: substantial endosteal cortical residues occurred in the cortical layer, which had a clearly porous appearance (Fig. 1C). In this study, based on the clinical guidelines of the JSOMR [14], patients with a severely eroded cortex were suspected to have osteoporosis.

### 2.3 Referral protocol to orthopedic department

When oral radiologists at our institution identified patients with suspected osteoporosis based on DPRs during routine diagnostic practice, they contacted the patients’ primary dentists and recommended referral to an orthopedic surgeon. Additional explanations about osteoporosis were provided verbally by oral radiologists upon request, with the support of photographic materials. All oral radiologists at our hospital were educated and trained in osteoporosis management and screening. Adequate interobserver agreement for osteoporosis screening using DPRs among the radiologists was previously confirmed [17].

### 2.4 BMD measurement and spinal fracture assessment

Diagnoses were based on DXA measurements. All referred patients underwent BMD assessment using DXA. Osteopenia was defined as a BMD T-score of between −2.5 and −1.0 at either the lumbar spine or femoral neck, according to the World Health Organization classification. Osteoporosis was defined as a BMD T-score of less than −2.5. The BMD was measured using a Lunar iDXA device (GE Healthcare Technologies, Inc., Chicago, IL, USA) at both the lumbar spine and femoral neck. Scanning parameters followed the hospital protocol: tube voltage and current were set at 76.0 kV and 3.0 mA for the lumbar spine and 76.0 kV and 0.75 mA for the femoral neck, respectively. An experienced orthopedic surgeon evaluated fractures of the thoracic and lumbar vertebrae using lateral spine radiographs.

### 2.5 Referral outcomes and prevalence analysis

We evaluated the overall referral rate of patients with suspected osteoporosis to orthopedic care; differences in referral rates between those who received an explanation from oral radiologists and those who did not; referral rates stratified by age group; and the prevalence of osteoporosis, osteopenia, and asymptomatic vertebral fractures detected on lateral radiographs among patients who visited the orthopedic department following referral. In addition, the proportion of referred patients with whom treatment was initiated, along with the types of medications prescribed, was evaluated.

### 2.6 Statistical analysis

Categorical variables are presented as N (%), and continuous variables as mean ± standard deviation (SD). Statistical analyses were performed using IBM SPSS Statistics for Windows (version 24.0; IBM Corp., Armonk, NY, USA). Differences in referral rates were analyzed using a two-sided chi-square test, with a significance level set at P < 0.05.

### 2.7 Ethical considerations

This study was approved by the Ethics Committee of Matsumoto Dental University (approval no. 0281). All procedures involving human participants were conducted in accordance with the ethical standards of the institutional and/or national research committee and with the 1964 Helsinki Declaration and its later amendments or comparable ethical standards. Written informed consent was obtained from all participants.

## 3 Results

DPRs were captured for 3237 female patients aged ≥ 50 for dental treatment at our institution during the study period. Of these patients, based on DPR findings, 362 (11.2%) were suspected of having osteoporosis. After excluding 34 patients with a history of orthopedic consultation for osteoporosis, 328 patients were included in the final analysis. Of these, 53 patients aged 59 to 89 visited the orthopedic department. The overall referral rate for orthopedic care, based on DPR findings, was 16.2% (53/328). The characteristics of the 53 referred patients and their treatment protocols are presented in Table 1.

**Table 1:** Characteristics of 53 patients referred for orthopedic care.

Variable	Mean ± SD or N (%)
Age, yrs	72.2 (7.3)
Lumbar spine (L2–L4) T-score	−1.9 (1.2)
Femoral neck T-score	−2.3 (0.7)
Total hip T-score	−2.0 (0.8)
Diagnosis based on DXA measurement	
Normal	1 (1.9)
Osteopenia	20 (37.7)
Osteoporosis	32 (60.3)
Vertebral fracture	
Thoracic vertebrae	2 (3.8)
Lumbar vertebrae	2 (3.8)
Both	1 (1.9)
Treatment for osteoporosis following referral	
No treatment	9 (17.0)
Vitamin D (active form)	24 (45.3)
SERM	10 (18.9)
Bisphosphonate	10 (18.9)

DXA, dual-energy X-ray absorptiometry; SD, standard deviation; SERM, selective estrogen receptor modulator.

Among the 53 referred patients, 32 were diagnosed with osteoporosis, 20 with osteopenia, and 5 (9.4%) had asymptomatic thoracic, lumbar, or combined vertebral fractures. Three of the nine patients who did not undergo treatment were diagnosed with osteoporosis; one of these three patients declined treatment due to a planned dental implant placement. Forty-four patients underwent treatment with osteoporosis medications after referral to an orthopedic surgeon and have continued therapy (Table 1): of the patients treated with bisphosphonates, nine were treated with alendronate and one with minodronate.

**Fig. 2:**
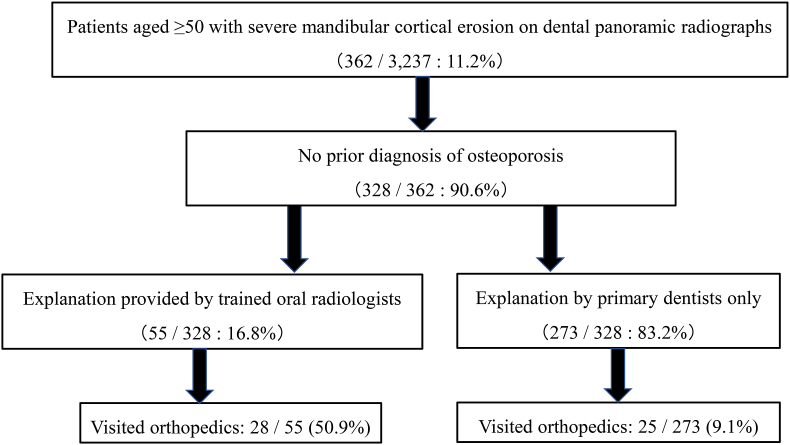
Flowchart of female dental patients aged ≥ 50 with severe mandibular cortical erosion on dental panoramic radiographs and their orthopedic referral outcomes. Patients were stratified by whether a trained oral radiologist or their primary dentist provided an explanation of osteoporosis and dental panoramic radiographic screening. Referral outcomes are also shown.

Among the study cohort, 55 (16.8%) patients requested additional information about osteoporosis and DPR-based screening from oral radiologists. Twenty-eight of these patients visited an orthopedic surgeon for further evaluation. Only 25 of the 273 patients who did not receive additional explanations visited an orthopedic surgeon (Fig. 2). Patients who received explanations from oral radiologists had significantly higher referral rates than those who did not (P < 0.001).

The age distribution of the patient population is shown in Table 2. Compared with other age groups, the proportion of patients with suspected osteoporosis without a prior orthopedic consultation was highest in the 60s age group. The age-specific referral rate to orthopedic surgeons was highest among those in their 60s. Forty-one (77.4%) of the fifty-three referred patients were in their 60s or 70s.

**Table 2:** Age distribution and osteoporosis-related findings of female patients who underwent dental panoramic radiography between February 2022 and October 2024.

Age group	50s	60s	70s	80s	90s	Total
All female patients	1053	859	906	364	55	3237
Suspected osteoporosis, N (%)	23 (2.2 %)	82 (9.5 %)	169 (18.7 %)	75 (20.6 %)	13 (23.6 %)	362 (11.2 %)
No prior osteoporosis diagnosis, N (%)	21 (91.3 %)	76 (92.7%)	153 (90.5 %)	66 (88.0 %)	12 (92.3 %)	328 (90.6 %)
Referred for orthopedic care, N (% of those without prior diagnosis)	3 (14.3 %)	18 (23.7 %)	23 (15.0 %)	9 (13.6 %)	0 (0 %)	53 (16.1 %)
DXA-based diagnosis in referred patients						
Normal	1	0	0	0	0	1
Osteopenia	0	6	9	5	0	20
Osteoporosis	2	12	14	4	0	32
Asymptomatic vertebral fracture	0	2	2	1	0	5

DXA, dual-energy X-ray absorptiometry.

## 4 Discussion

In this prospective study, the rate of referral to orthopedic care based on DPR findings was 16.2%, a similar rate to that found in our pilot study, in which only 23% of identified postmenopausal women were referred for DXA evaluation [16]. This low referral rate reflects the broader context in Japan, where the average screening rate for osteoporosis is only 5.5%; significant regional disparities exist, with rates ranging from 0.3% to 14.0% [5]. Municipalities with higher screening rates have been associated with reduced rates of surgery to treat femoral fractures and long-term care needs, emphasizing the importance of promoting screening among middle-aged and older women [5].

Recently, Yu and Wang pointed out in their review that osteoporotic patients who sustain fractures tend to exhibit progression of periodontitis, leading to an elevated risk of tooth loss [18]. This finding implies that patients visiting dental clinics are likely to be at higher risk of having osteoporosis. Therefore, if dentists could screen for osteoporosis using DPRs, the overall screening rate could be substantially increased. In addition, we emphasize that opportunistic screening using DPRs may facilitate earlier detection, particularly since approximately 5 million DPRs are taken annually in Japan among individuals aged 65 years and older [6]. Collaboration between dentists and physicians could further enhance timely referrals and improve the national screening rate.

Barriers to osteoporosis screening have been described: In a national survey, Choksi et al. found that patient nonadherence, physician concerns about cost, limited consultation time, and low prioritization were major obstacles [19]. Similarly, Sato et al. reported that only 15% of Japanese women aged ≥ 50 perceived themselves to be at risk for osteoporosis, and this perception was primarily associated with family history, not clinical risk factors such as smoking or glucocorticoid use [20]. These findings suggest that the low awareness of risk among both patients and providers is a significant contributor to under-screening.

In the present study, the referral rates of patients who received information from trained oral radiologists were significantly higher than those who did not. This suggests that communication from informed dental professionals can enhance patients’ willingness to seek further evaluation. Since DPRs are routinely captured in general dental practice, incorporating osteoporosis risk assessment offers a practical, low-burden approach to opportunistic screening. Educating general dental practitioners on osteoporosis-related findings and referral criteria could improve early identification and referral rates. We previously demonstrated that even minimal training enabled dental students to outperform the Osteoporosis Self-Assessment Tool for Asians in identifying at-risk patients using DPRs [21]; this supports the integration of osteoporosis education into dental curricula.

Among referred patients, 98.1% were diagnosed with osteopenia or osteoporosis, and 9.4% had asymptomatic vertebral fractures. Most of these patients were in their 60s or 70s, suggesting that DPR-based screening is especially useful for identifying women aged 60 to 79 who should be evaluated by orthopedic specialists. Although 44 referred patients initiated osteoporosis treatment (24 with vitamin D, 10 with selective estrogen receptor modulator, and 10 with bisphosphonates) and have remained adherent to therapy to date, 9 patients did not initiate treatment. One patient, who had a femoral neck T-score of −3.2, declined treatment due to concerns related to planned dental implant placement. This reflects the ongoing clinical dilemma regarding the perceived risk of medication-related osteonecrosis of the jaw associated with antiresorptive therapy. Although some studies advise caution [22], the International Task Force on Osteonecrosis of the Jaw suggests that patients with osteoporosis who are undergoing dental implant procedures need not discontinue antiresorptive therapy [23]. Importantly, interruption of therapy, particularly with agents such as denosumab, may increase the risk of fragility fractures. Therefore, multidisciplinary collaboration among dentists, physicians, and orthopedic specialists, coupled with accurate sharing of information and patient education, is essential to support evidence-based decision-making and prevent inappropriate discontinuation of treatment.

This study had several limitations. First, the participants were female patients who visited a university hospital for dental treatment and may not reflect the general population of adults aged ≥ 50. The participants may have been more health-conscious, yet the referral rate remained low. The referral rate in the general population, particularly among women who do not routinely visit dental clinics, may be even lower. Further studies including community-based participants and male patients are warranted to confirm the generalizability of our findings, although there is currently no evidence regarding DPR-based screening in men [14]. Future research should aim to establish such evidence.

Second, the dentists at our institution had limited knowledge about osteoporosis, which may have contributed to missed referral opportunities. In contrast, Kure City has implemented a successful multidisciplinary model involving physicians, dentists, and public health officials to enhance osteoporosis care [24]. Finally, we were unable to assess the reasons why many referred patients did not follow through with orthopedic consultations. Understanding these reasons could guide future strategies to improve patient adherence and referral outcomes.

## 5 Conclusions

In this study, only 16.2% of patients with suspected osteoporosis, identified by a DPR finding of severe mandibular cortical erosion, consulted an orthopedic surgeon. Of those referred, 60.4% were diagnosed with osteoporosis, 37.7% with osteopenia, and five had asymptomatic vertebral fractures. Patients who received explanations about osteoporosis and DPR screening from trained oral radiologists were more likely to visit orthopedics than those who did not. These findings indicate that expert-led explanations significantly boost referral rates, highlighting the importance of dentist-led education in promoting osteoporosis screening. Future research should explore the long-term impact of dentist-led education on osteoporosis management outcomes and identify strategies to improve referral and treatment rates in broad populations.

## CRediT author statement

**Noriyuki Sugino**: Conceptualization, Methodology, Validation, Formal analysis and investigation, Software, Writing - review and editing, Resources. **Hiroko Kuroiwa**: Conceptualization, Methodology, Writing - review and editing, Resources. **Hizuru Osanai**: Conceptualization, Methodology, Validation, Writing - review and editing, Resources. **Hirokazu Kobayashi**: Conceptualization, Project administration, Writing - review and editing, Resources, **Daisuke Higuchi**: Conceptualization, Project administration, Writing - review and editing. **Nobuyuki Udagawa**: Conceptualization, Project administration, Writing - review and editing, Supervision. **Akira Taguchi**: Conceptualization, Methodology, Validation, Project administration, Formal analysis and investigation, Software, Writing - original draft preparation, Funding acquisition, Supervision. **Shinichiro Yamada**: Methodology, Validation, Writing - review and editing, Resources. **Kozue Mori**: Methodology, Validation, Writing - review and editing, Resources.

## Declaration of generative AI in scientific writing

The authors did not use generative AI or AI-assisted technologies in the preparation of this manuscript.

## Conflicts of interest

Akira Taguchi received lecture fees from MEDIA Co., Ltd., Asahi Kasei Pharma Corp., 10.13039/501100002973Daiichi Sankyo Co., Ltd., 10.13039/100002429Amgen Inc., and Taisho Pharmaceutical Co., Ltd. Noriyuki Sugino, Hiroko Kuroiwa, Hizuru Osanai, Shinichiro Yamada, Kozue Mori, Hirokazu Kobayashi, Daisuke Higuchi, and Nobuyuki Udagawa declare no competing interests.
